# Supplementary material for: Evaluation of the Impact of Population Management on the Genetic Parameters of Selected Spiral-Horned Antelopes
Source: Biology (Basel). 2024 Feb 7;13(2):104. doi: 10.3390/biology13020104 (PMC10886411; doi:10.3390/biology13020104)
Supplement: Supplementary file 1 [file biology-13-00104-s001.zip › biology-2825491-supplementary.pdf]

Table S1. Overview of number of samples analyzed in this study, categorized with regards to the taxa and institutions of samples collection.

Institution	Country	Number of samples
**Mountain bongo**		**13**
GaiaZOO Kerkrade	Netherlands	1
Knowsley Safari	United Kingdom	1
Safari de Peaugres	France	1
Safari Park Dvůr Králové	Czechia	8
unknown	-	2
**Nyala**		**65**
Africa Alive Zoological Reserve	United Kingdom	1
Edinburgh Zoo	United Kingdom	7
Hellabrunn Zoo Munich	Germany	1
Jihlava Zoo	Czechia	3
Marwell Zoo	United Kingdom	3
Pilsen Zoo	Czechia	10
Safari Park Dvůr Králové	Czechia	28
Vienna Zoo	Austria	7
Zoo Hannover	Germany	5
**Sitatunga**		**32**
Africa Alive Zoological Reserve	United Kingdom	1
Exmoor Zoo	United Kingdom	2
Knowsley Safari	United Kingdom	1
Safari Park Dvůr Králové	Czechia	19
Serengeti-Park Hodenhagen	Germany	1
Zoo African Safari Plaisance-du-Touch	France	1
unknown	-	7
**Lesser kudu**		**42**
Hannover Zoo	Germany	2
Safari Park Dvůr Králové	Czechia	35
Serengeti-Park Hodenhagen	Germany	1
unknown	-	4
**Greater kudu**[1]		**31**
GaiaZOO Kerkrade	Netherlands	2
Hellabrunn Zoo Munich	Germany	2
Pilsen Zoo	Czechia	2
Safari Park Dvůr Králové	Czechia	16
Serengeti-Park Hodenhagen	Germany	1
Zoo Boissière du Doré	France	1
Zoo La Palmyre	France	2
unknown	-	5
**Common eland**[2]		**36**
Hellabrunn Zoo Munich	Germany	2
Knowsley Safari	United Kingdom	5
Safari Park Dvůr Králové	Czechia	16
University Farm Estate Lány	Czechia	9
Vienna Zoo	Austria	2
Zoo African Safari Plaisance-du-Touch	France	1
Zoo Hannover	Germany	1

[1] samples were equally collected from Rotterdam Zoo (Netherlands) but were not used due to failure to amplify
[2] samples were equally collected from Givskud Zoo (Denmark) but were not used due to failure to amplify

Table S2. Selected panel of 10 microsatellite loci, divided into two multiplexes, including sequences of their forward and reverse primers, used fluorescent dye label, length of fragments detected in this study, and original reference.

	Forward primer<br>Reverse primer	Dye label	Detected size in base pairs	Reference
**Multiplex 1**				
BL42	CAAGGTCAAGTCCAAATGCC<br>GCATTTTTGTGTTAATTTCATGC	FAM	279-315	[96]
BRR	TGCTCTTACCTGCCACACCCG<br>CCCTCTTCTCACCCCCCAAAAC	NED	233-261	[97]
CSRM60	AAGATGTGATCCAAGAGAGAGGCA<br>AGGACCAGATCGTGAAAGGCA	FAM	83-129	[98]
ETH10	GTTCAGGACTGGCCCTGCTAACA<br>CCTCCAGCCCACTTTCTCTTCT	FAM	200-226	[99]
ETH225	GATCACCTTGCCACTATTTCCT<br>ACATGACAGCCAGCTGCTACT	NED	131-165	[100]
INRA011	CGAGTTTCTTTCCTCGTGGTAGGC<br>GCTCGGCACATCTTCCTTAGCAACT	VIC	203-239	[101]
**Multiplex 2**				
BM4505	TTATCTTGGCTTCTGGGTGC<br>ATCTTCACTTGGGATGCAGG	FAM	239-291	[96]
CSSM42	GGGAAGGTCCTAACTATGGTTGAG<br>ACCCTCACTTCTAACTGCATTG	PET	156-222	[102]
INRA107	TCCCAGATACAGATGCAACAG<br>GGAGAGCCGAGGGCTTCAGC	FAM	148-180	[103]
SPS113	CCTCCACACAGGCTTCTCTGACTT<br>CCTAACTTGCTTGAGTTATTGC	PET	127-155	[104]

Table S3. List of sequences from the National Center for Biotechnology Information (NCBI) Nucleotide database [56] used in this study, including their accession numbers, countries of origin, and source publications

Accession number	Country of origin	Reference
**Mountain bongo**		
EU040245.1	Kenya	[68]
EU040246.1	Kenya	[68]
JN632703.1	Congo	[105]
**Nyala**		
AY530163.1	Malawi	[106]
AY530164.1	Malawi	[106]
AY530165.1	Malawi	[106]
AY530166.1	Malawi	[106]
AY530167.1	Malawi	[106]
AY530168.1	Malawi	[106]
AY530169.1	Malawi	[106]
AY530170.1	Malawi	[106]
AY530171.1	Malawi	[106]
AY530172.1	Malawi	[106]
AY530173.1	Mozambique	[106]
AY530174.1	South Africa	[106]
AY530175.1	South Africa	[106]
AY530176.1	South Africa	[106]
AY530177.1	South Africa	[106]
AY530178.1	Zimbabwe	[106]
AY530179.1	South Africa	[106]
AY530180.1	South Africa	[106]
AY530181.1	South Africa	[106]
JN632702.1	South Africa	[105]
**Sitatunga**		
EF536357.1	Gabon	[105]
FJ823281.1	Congo	[107]
FJ823282.1	Cameroon	[107]
FJ823283.1	Gabon	[107]
FJ823284.1	Gabon	[107]
FJ823285.1	Gabon	[107]
FJ823286.1	Gabon	[107]
MH792168.1	unknown	[108]
**Lesser kudu**		
AF301711.1	Tanzania	[109]
AF301712.1	Tanzania	[109]
EF536356.1	Tanzania	[105]
HG931349.1	captive - Kenya[1]	[65]
HG931350.1	captive - Somalia[1]	[65]
HG931351.1	captive - Kenya[1]	[65]
HG931352.1	unknown	[65]
HG931353.1	captive - Kenya[1]	[65]
HG931354.1	captive - Kenya[1]	[65]
HG931355.1	captive - Kenya[1]	[65]
HG931356.1	captive - Kenya[1]	[65]
HG931357.1	captive - Kenya[1]	[65]

HG931358.1	captive - Kenya[1]	[65]
HG931359.1	captive - Kenya[1]	[65]
HG931360.1	captive - Kenya[1]	[65]
HG931361.1	captive - Kenya[1]	[65]
HG931362.1	captive - Kenya[1]	[65]
HG931363.1	captive - Kenya[1]	[65]
HG931364.1	captive - Kenya[1]	[65]
HG931365.1	captive - Kenya[1]	[65]
HG931366.1	captive - Kenya[1]	[65]
HG931367.1	captive - Kenya[1]	[65]
HG931368.1	captive - Kenya[1]	[65]
HG931369.1	captive - Kenya[1]	[65]
HG931371.1	captive - Kenya[1]	[65]
HG931372.1	captive - Kenya[1]	[65]
HG931373.1	captive - Kenya[1]	[65]
HG931374.1	captive - Somalia[1]	[65]
HG931375.1	captive - Somalia[1]	[65]
HG931376.1	captive - Somalia[1]	[65]
HG931377.1	captive - Kenya[1]	[65]
HG931378.1	captive - Kenya[1]	[65]
HG931379.1	captive - Kenya[1]	[65]
HG931380.1	unknown	[65]
HG931381.1	unknown	[65]
HG931382.1	unknown	[65]
HG931383.1	unknown	[65]
HG931384.1	unknown	[65]
HG931385.1	unknown	[65]
HG931386.1	unknown	[65]
HG931387.1	unknown	[65]
HG931388.1	unknown	[65]
HG931389.1	unknown	[65]
HG931390.1	unknown	[65]
HG931391.1	unknown	[65]
HG931392.1	unknown	[65]
HG931393.1	captive - Kenya[1]	[65]
HG931394.1	captive - Kenya[1]	[65]
HG931395.1	captive - Kenya[1]	[65]
HG931396.1	captive - Kenya[1]	[65]
HG931397.1	captive - Kenya[1]	[65]
HG931398.1	unknown	[65]
KY628399.1	unknown	unpublished
**Greater kudu**		
AF301621.1	Tanzania	[109]
AF301622.1	Tanzania	[109]
AF301623.1	Tanzania	[109]
AF301624.1	Tanzania	[109]
AF301625.1	Tanzania	[109]
AF301626.1	Tanzania	[109]
AF301627.1	Tanzania	[109]
AF301628.1	Tanzania	[109]
AF301629.1	Tanzania	[109]

AF301630.1	Botswana	[109]
AF301631.1	Botswana	[109]
AF301632.1	Botswana	[109]
AF301633.1	Botswana	[109]
AF301634.1	Botswana	[109]
AF301635.1	Botswana	[109]
AF301636.1	Botswana	[109]
AF301637.1	Botswana	[109]
AF301638.1	Botswana	[109]
AF301639.1	Botswana	[109]
AF301640.1	Botswana	[109]
AF301641.1	Botswana	[109]
AF301642.1	Botswana	[109]
AF301643.1	Botswana	[109]
AF301644.1	Namibia	[109]
AF301645.1	Namibia	[109]
AF301646.1	Namibia	[109]
AF301647.1	Namibia	[109]
AF301648.1	Namibia	[109]
AF301649.1	Namibia	[109]
AF301650.1	Namibia	[109]
AF301651.1	Namibia	[109]
AF301652.1	Namibia	[109]
AF301653.1	Namibia	[109]
AF301654.1	Namibia	[109]
AF301655.1	Namibia	[109]
AF301656.1	Namibia	[109]
AF301657.1	Namibia	[109]
AF301658.1	Namibia	[109]
AF301659.1	Namibia	[109]
AF301660.1	Namibia	[109]
AF301661.1	Namibia	[109]
AF301662.1	Namibia	[109]
AF301663.1	Namibia	[109]
AF301664.1	Tanzania	[109]
AF301665.1	Tanzania	[109]
AF301666.1	Tanzania	[109]
AF301667.1	Tanzania	[109]
AF301668.1	Tanzania	[109]
AF301669.1	Tanzania	[109]
AF301670.1	Tanzania	[109]
AF301671.1	Tanzania	[109]
AF301672.1	Tanzania	[109]
AF301673.1	Zambia	[109]
AF301674.1	Namibia	[109]
AF301675.1	Namibia	[109]
AF301676.1	Zambia	[109]
AF301677.1	Zambia	[109]
AF301678.1	Zambia	[109]
AF301679.1	Tanzania	[109]
AF301680.1	Tanzania	[109]
AF301681.1	Tanzania	[109]

AF301682.1	Tanzania	[109]
AF301683.1	Tanzania	[109]
AF301684.1	Tanzania	[109]
AF301685.1	Tanzania	[109]
AF301686.1	Tanzania	[109]
AF301687.1	Tanzania	[109]
AF301688.1	Namibia	[109]
AF301689.1	Namibia	[109]
AF301690.1	Namibia	[109]
AF301691.1	Namibia	[109]
AF301692.1	Namibia	[109]
AF301693.1	Namibia	[109]
AF301694.1	Namibia	[109]
AF301695.1	Botswana	[109]
AF301696.1	Botswana	[109]
AF301697.1	Namibia	[109]
AF301698.1	Namibia	[109]
AF301699.1	Namibia	[109]
AF301700.1	South Africa	[109]
AF301701.1	Zimbabwe	[109]
AF301702.1	Zimbabwe	[109]
AF301703.1	Zimbabwe	[109]
AF301704.1	Zimbabwe	[109]
AF301705.1	Zimbabwe	[109]
AF301706.1	Zimbabwe	[109]
AF301707.1	Tanzania	[109]
AF301708.1	Tanzania	[109]
AF301709.1	Tanzania	[109]
JN632708.1	Tanzania	[105]
MG839216.1	unknown	[66]
MG839217.1	unknown	[66]
OK642751.1	South Africa	[110]
OK642752.1	South Africa	[110]
OK642753.1	South Africa	[110]
OK642754.1	South Africa	[110]
OK642755.1	South Africa	[110]
OK642756.1	South Africa	[110]
OK642757.1	South Africa	[110]
OK642758.1	South Africa	[110]
OK642759.1	South Africa	[110]
OK642760.1	South Africa	[110]
OK642761.1	South Africa	[110]
OK642762.1	South Africa	[110]
OK642763.1	South Africa	[110]
OK642764.1	South Africa	[110]
OK642765.1	South Africa	[110]
OK642766.1	South Africa	[110]
OK642767.1	South Africa	[110]
OK642768.1	South Africa	[110]
OK642769.1	South Africa	[110]
OK642770.1	South Africa	[110]
OK642771.1	South Africa	[110]

OK642772.1	South Africa	[110]
OK642773.1	South Africa	[110]
OK642774.1	South Africa	[110]
OK642775.1	South Africa	[110]
OK642776.1	South Africa	[110]
**Common eland**		
GQ388117.1	unknown	[111]
GQ388118.1	unknown	[111]
GQ388119.1	Ethiopia	[111]
GQ388120.1	Ethiopia	[111]
GQ388121.1	Ethiopia	[111]
GQ388122.1	Ethiopia	[111]
GQ388123.1	Kenya	[111]
GQ388124.1	Kenya	[111]
GQ388125.1	Kenya	[111]
GQ388126.1	Tanzania	[111]
GQ388127.1	Tanzania	[111]
GQ388128.1	Tanzania	[111]
GQ388129.1	Tanzania	[111]
GQ388130.1	Tanzania	[111]
GQ388131.1	Tanzania	[111]
GQ388132.1	Tanzania	[111]
GQ388133.1	Tanzania	[111]
GQ388134.1	Tanzania	[111]
GQ388135.1	Tanzania	[111]
GQ388136.1	Tanzania	[111]
GQ388137.1	Tanzania	[111]
GQ388138.1	Tanzania	[111]
GQ388139.1	Tanzania	[111]
GQ388140.1	Tanzania	[111]
GQ388141.1	Tanzania	[111]
GQ388142.1	Tanzania	[111]
GQ388143.1	Tanzania	[111]
GQ388144.1	Tanzania	[111]
GQ388145.1	Tanzania	[111]
GQ388146.1	Tanzania	[111]
GQ388147.1	Tanzania	[111]
GQ388148.1	Tanzania	[111]
GQ388149.1	Tanzania	[111]
GQ388150.1	Tanzania	[111]
GQ388151.1	Tanzania	[111]
GQ388152.1	Tanzania	[111]
GQ388153.1	Tanzania	[111]
GQ388154.1	Tanzania	[111]
GQ388155.1	Tanzania	[111]
GQ388156.1	Tanzania	[111]
GQ388157.1	Tanzania	[111]
GQ388158.1	Tanzania	[111]
GQ388159.1	Tanzania	[111]
GQ388160.1	Tanzania	[111]
GQ388161.1	Tanzania	[111]

GQ388162.1	Tanzania	[111]
GQ388163.1	Tanzania	[111]
GQ388164.1	Tanzania	[111]
GQ388165.1	Tanzania	[111]
GQ388166.1	Tanzania	[111]
GQ388167.1	Tanzania	[111]
GQ388168.1	Tanzania	[111]
GQ388169.1	Tanzania	[111]
GQ388170.1	Uganda	[111]
GQ388171.1	Uganda	[111]
GQ388172.1	Uganda	[111]
GQ388173.1	Uganda	[111]
GQ388174.1	Uganda	[111]
GQ388175.1	Uganda	[111]
GQ388176.1	Uganda	[111]
GQ388177.1	Uganda	[111]
GQ388178.1	Uganda	[111]
GQ388179.1	Uganda	[111]
GQ388180.1	Uganda	[111]
GQ388181.1	Botswana	[111]
GQ388182.1	Botswana	[111]
GQ388183.1	Botswana	[111]
GQ388184.1	Botswana	[111]
GQ388185.1	Botswana	[111]
GQ388186.1	Botswana	[111]
GQ388187.1	Botswana	[111]
GQ388188.1	Botswana	[111]
GQ388189.1	Botswana	[111]
GQ388190.1	Botswana	[111]
GQ388191.1	Namibia	[111]
GQ388192.1	Namibia	[111]
GQ388193.1	Namibia	[111]
GQ388194.1	Namibia	[111]
GQ388195.1	Namibia	[111]
GQ388196.1	Namibia	[111]
GQ388197.1	Namibia	[111]
GQ388198.1	Namibia	[111]
GQ388199.1	Namibia	[111]
GQ388200.1	Namibia	[111]
GQ388201.1	Namibia	[111]
GQ388202.1	Namibia	[111]
GQ388203.1	Namibia	[111]
GQ388204.1	Namibia	[111]
GQ388205.1	Namibia	[111]
GQ388206.1	Namibia	[111]
GQ388207.1	Namibia	[111]
GQ388208.1	Namibia	[111]
GQ388209.1	Namibia	[111]
GQ388210.1	Namibia	[111]
GQ388211.1	Namibia	[111]
GQ388212.1	South Africa	[111]
GQ388213.1	Zambia	[111]

GQ388214.1	Zambia	[111]
GQ388215.1	Zambia	[111]
GQ388216.1	Zambia	[111]
GQ388217.1	Zambia	[111]
GQ388218.1	Zimbabwe	[111]
GQ388219.1	Zimbabwe	[111]
GQ388220.1	Zimbabwe	[111]
GQ388221.1	Zimbabwe	[111]
GQ388222.1	Zimbabwe	[111]
GQ388223.1	Zimbabwe	[111]
GQ388224.1	Zimbabwe	[111]
GQ388225.1	Zimbabwe	[111]
GQ388226.1	Zimbabwe	[111]
GQ388227.1	Zimbabwe	[111]
GQ388228.1	Zimbabwe	[111]
GQ388229.1	Zimbabwe	[111]
GQ388230.1	Zimbabwe	[111]
GQ388231.1	Zimbabwe	[111]
GQ388232.1	Zimbabwe	[111]
GQ388233.1	Zimbabwe	[111]
GQ388234.1	Zimbabwe	[111]
GQ388235.1	Zimbabwe	[111]
GQ388236.1	Zimbabwe	[111]
GQ388237.1	Zimbabwe	[111]
GQ388238.1	Zimbabwe	[111]
JN632704.1	Tanzania	[105]
MG839218.1	unknown	[66]
MG839219.1	unknown	[66]
MG839220.1	unknown	[66]

[1] sequences of captive animals with origin determined using studbook data, which were used due to low availability of sequences of wild individuals in lesser kudu

Table S4. Accession numbers of sequences from this study

Sample code	Accession number
**Mountain Bongo**	
BO01	PP061542
BO02	PP061543
BO03	PP061544
BO04	PP061545
BO05	PP061546
BO06	PP061547
BO07	PP061548
BO08	PP061549
**Nyala**	
NY01	PP061550
NY02	PP061551
NY03	PP061552
NY04	PP061553
NY05	PP061554
NY06	PP061555
NY07	PP061556
NY08	PP061557
NY09	PP061558
**Sitatunga**	
SI01	PP061559
SI02	PP061560
SI03	PP061561
SI04	PP061562
SI05	PP061563
SI06	PP061564
SI07	PP061565
SI08	PP061566
SI09	PP061567
SI10	PP061568
SI11	PP061569
SI12	PP061570
SI13	PP061571
**Lesser kudu**	
LK01	PP061572
LK02	PP061573
LK03	PP061574
LK04	PP061575
LK05	PP061576
LK06	PP061577
LK07	PP061578
LK08	PP061579
**Greater kudu**	
GK01	PP061580
GK02	PP061581
GK03	PP061582
GK04	PP061583
GK05	PP061584
GK06	PP061585

GK07	PP061586
GK08	PP061587
GK09	PP061588
GK10	PP061589
GK11	PP061590
GK12	PP061591
GK13	PP061592
GK14	PP061593
GK15	PP061594
GK16	PP061595
GK17	PP061596
**Common eland**	
CE01	PP061597
CE02	PP061598
CE03	PP061599
CE04	PP061600
CE05	PP061601
CE06	PP061602
CE07	PP061603
CE08	PP061604
CE09	PP061605
CE10	PP061606
CE11	PP061607
CE12	PP061608
CE13	PP061609
CE14	PP061610
CE15	PP061611
CE16	PP061612
CE17	PP061613
CE18	PP061614

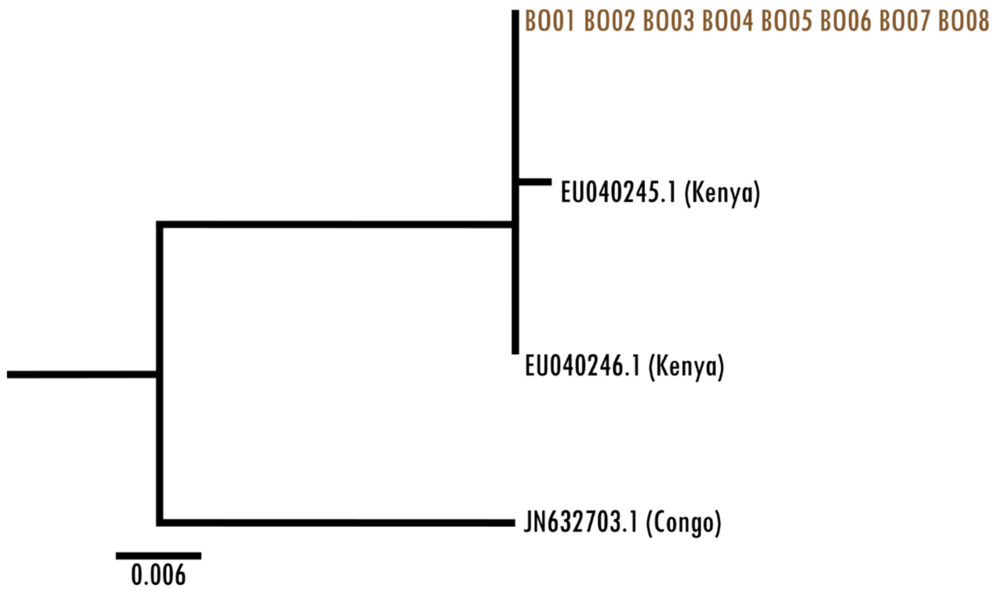

Figure S1. Neighbor-joining phylogenetic tree for mountain bongo. Samples from this study are shown in brown and identified by their unique codes (see Table S4). Sequences from the NCBI nucleotide database are referred to by their accession numbers, and their country of origin is indicated in brackets.

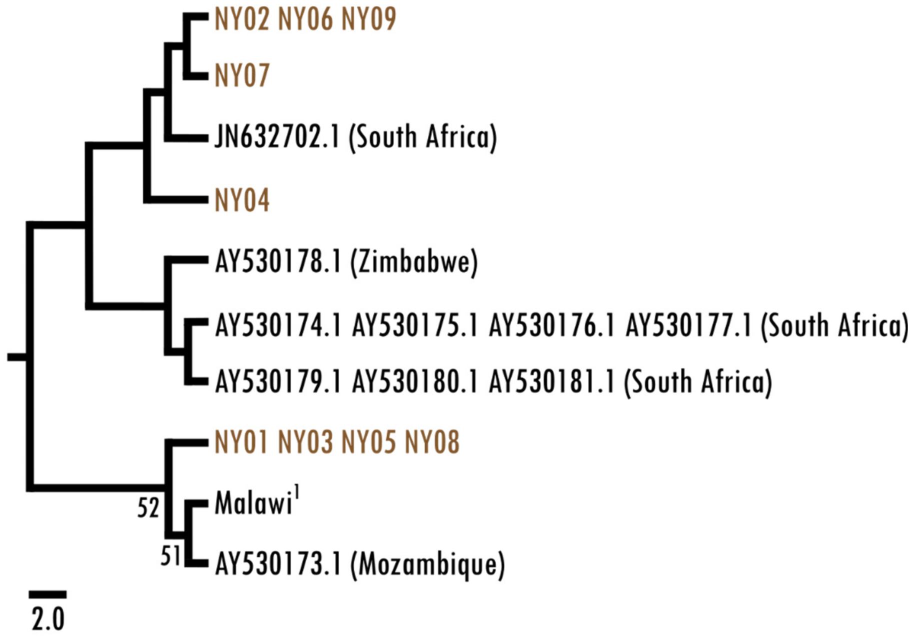

[1] AY530163.1 AY530164.1 AY530165.1 AY530166.1 AY530167.1 AY530168.1 AY530169.1 AY530170.1 AY530171.1 AY530172.1

Figure S2. Neighbor-joining phylogenetic tree for nyala. Samples from this study are shown in brown and identified by their unique codes (see Table S4). Sequences from the NCBI nucleotide database are referred to by their accession numbers, and their country of origin is indicated in brackets.

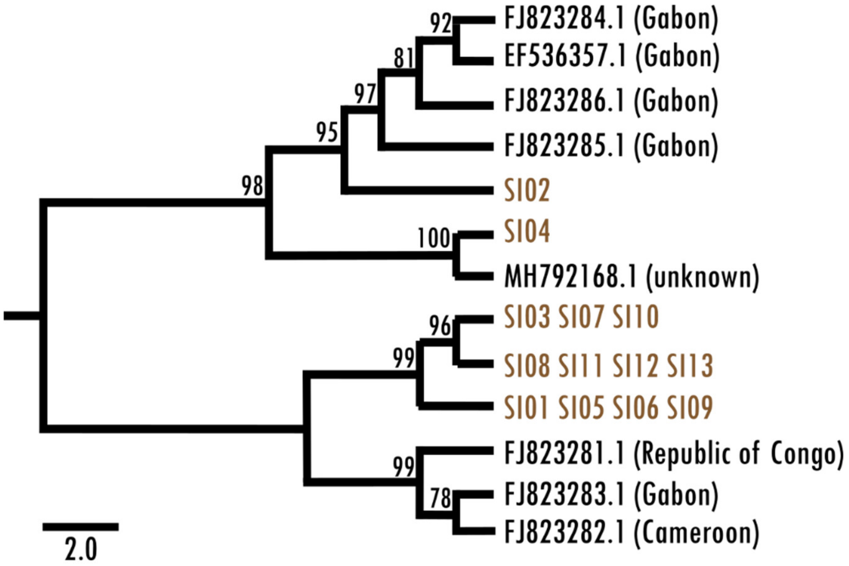

Figure S3. Neighbor-joining phylogenetic tree for sitatunga. Samples from this study shown in brown and identified by their unique codes (see Table S4). Sequences from the NCBI nucleotide database are referred to by their accession numbers, and their country of origin is indicated in brackets.

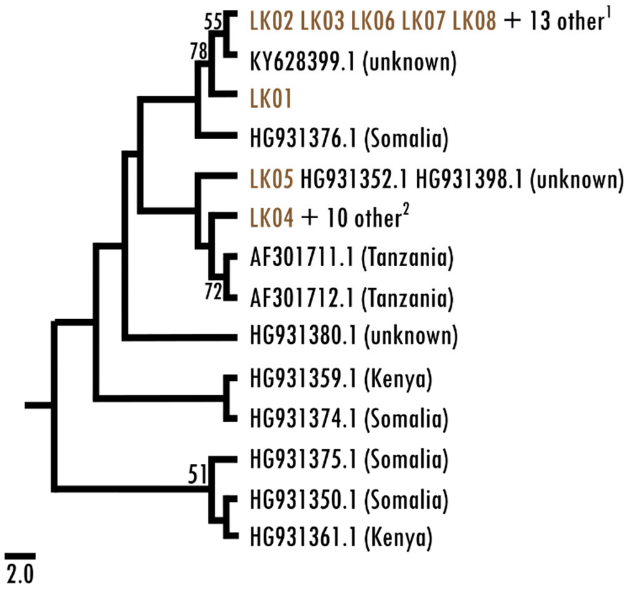

[1] HG931351.1 HG931357.1 HG931358.1 HG931360.1 HG931362.1 HG931363.1 HG931364.1 HG931365.1 HG931366.1 HG931367.1 HG931369.1 HG931371.1 HG931378.1 (Kenya)

[2] HG931349.1 HG931353.1 HG931354.1 HG931355.1 HG931356.1 HG931372.1 HG931373.1 (Kenya) HG931381.1 HG931386.1 HG931388.1 (unknown) EF536356.1 (Tanzania)

[3] HG931380.1 HG931382.1 HG931383.1 HG931384.1 HG931385.1 HG931387.1 HG931389.1 HG931390.1 HG931391.1HG931392.1

[4] HG931359.1 HG931368.1 HG931377.1 HG931393.1 HG931394.1 HG931395.1 HG931396.1 HG931397.1 HG931379.1

Figure S4. Neighbor-joining phylogenetic tree for lesser kudu. Samples from this study are shown in brown and identified by their unique codes (see Table S4). Sequences from the NCBI nucleotide database are referred to by their accession numbers, and their country of origin is indicated in brackets.

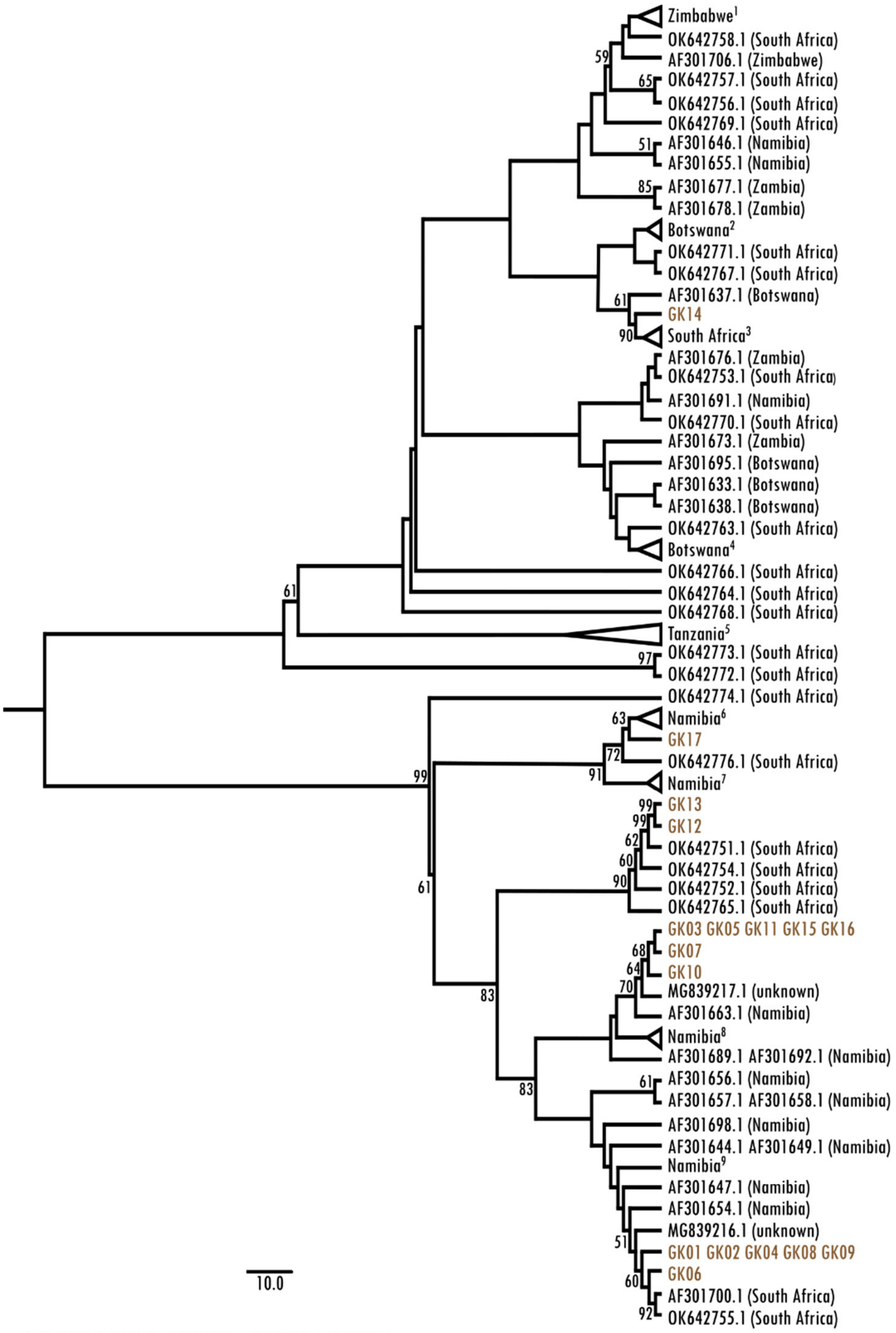

[1] AF301701.1 AF301702.1 AF301703.1 AF301704.1 AF301705.1
[2] AF301632.1 AF301635.1 AF301639.1 AF301640.1 AF301641.1 AF301642.1 AF301643.1
[3] OK642759.1 OK642760.1 OK642761.1 OK642762.1 OK642775.1
[4] AF301630.1 AF301631.1 AF301634.1 AF301636.1 AF301696.1
[5] AF301621.1 AF301622.1 AF301623.1 AF301624.1 AF301625.1 AF301626.1 AF301627.1 AF301628.1 AF301629.1 AF301664.1 AF301665.1 AF301666.1 AF301667.1 AF301668.1 AF301669.1 AF301670.1 AF301671.1 AF301672.1 AF301679.1 AF301680.1 AF301681.1 AF301682.1 AF301683.1 AF301684.1 AF301685.1 AF301686.1 AF301687.1 AF301707.1 AF301708.1 AF301709.1 JN632708.1
[6] AF301645.1 AF301648.1 AF301650.1 AF301652.1 AF301653.1
[7] AF301659.1 AF301660.1 AF301661.1 AF301674.1
[8] AF301662.1 AF301675.1 AF301697.1 AF301699.1
[9] AF301651.1 AF301688.1 AF301690.1 AF301693.1 AF301694.1

Figure S5. Neighbor-joining phylogenetic tree for greater kudu. Samples from this study are shown in brown and identified by their unique codes (see Table S4). Sequences from the NCBI nucleotide database are referred to by their accession numbers, and their country of origin is indicated in brackets.

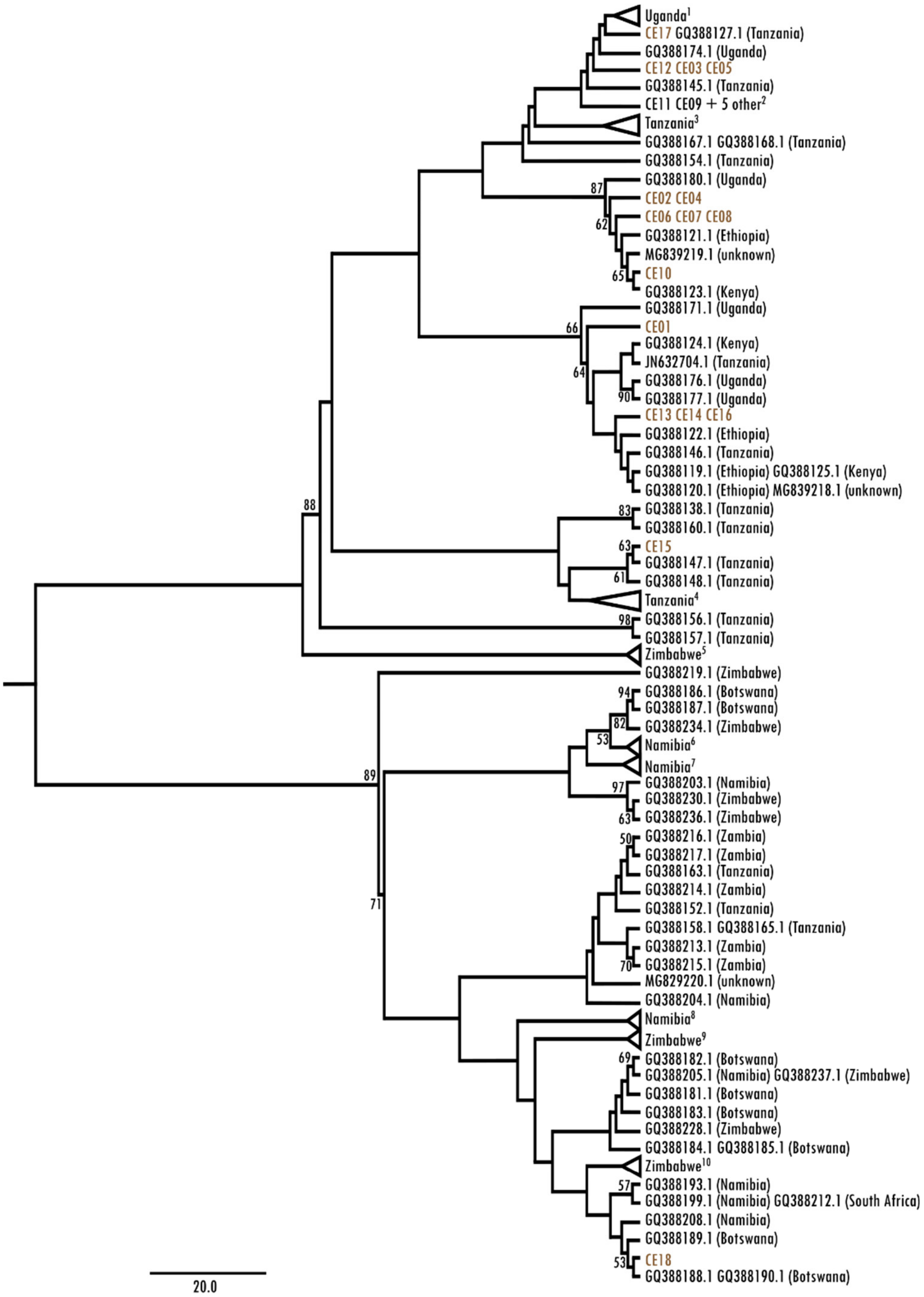

[1] GQ388170.1 GQ388172.1 GQ388173.1 GQ388175.1 GQ388178.1 GQ388179.1

[2] GQ388117.1 GQ388118.1 (UNK - Denmark) GQ388141.1 GQ388155.1 GQ388164.1 (Tanzania)

[3] GQ388126.1 GQ388130.1 GQ388135.1 GQ388136.1 GQ388139.1 GQ388140.1 GQ388142.1 GQ388144.1 GQ388149.1 GQ388151.1 GQ388153.1

[4] GQ388161.1 GQ388128.1 GQ388129.1 GQ3888131.1 GQ388132.1 GQ388133.1 GQ388134.1 GQ388137.1 GQ388143.1 GQ388150.1 GQ388156.1 GQ388157.1 GQ388159.1 GQ388162.1 GQ388166.1 GQ388169.1

[5] GQ388221.1 GQ388226.1 GQ388235.1

[6] GQ388194.1 GQ388207.1 GQ388211.1

[7] GQ388191.1 GQ388192.1 GQ388195.1 GQ388196.1 GQ388197.1 GQ388198.1 GQ388200.1 GQ388201.1 GQ388202.1

[8] GQ388206.1 GQ388209.1 GQ3882010.1

[9] GQ388220.1 GQ388222.1 GQ388225.1 GQ388231.1

[10] GQ388218.1 GQ388223.1 GQ338224.1 GQ388227.1 GQ388229.1 GQ388232.1 GQ388233.1 GQ388238.1

Figure S6. Neighbor-joining phylogenetic tree for common eland. Samples from this study are shown in brown and identified by their unique codes (see Table S4). Sequences from the NCBI nucleotide database are referred to by their accession numbers, and their country of origin is indicated in brackets.

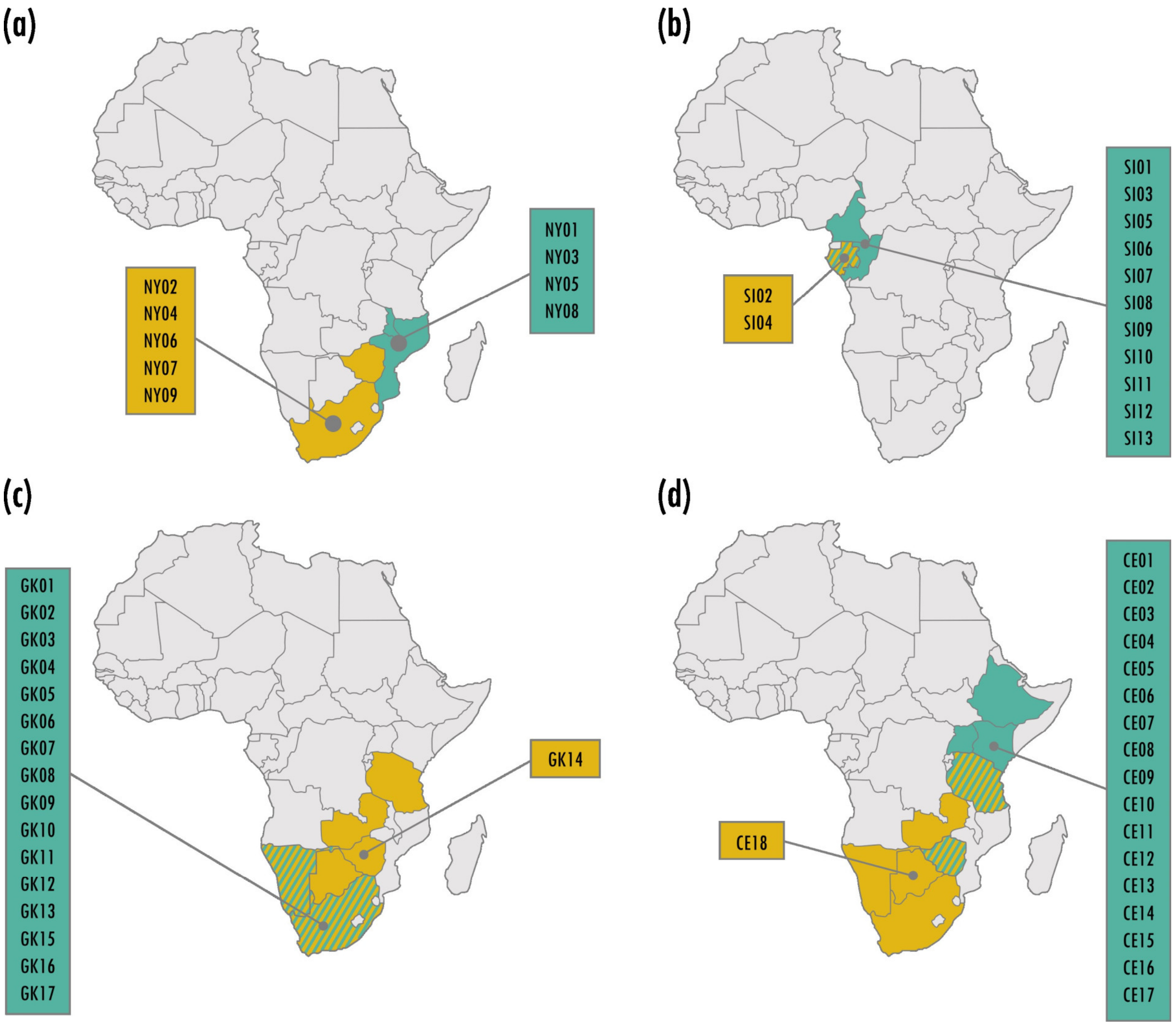

Figure S7. Overview of geographic distribution of genetically distinct clades in (a) nyala, (b) sitatunga, (c) greater kudu, (d) common eland, created based on the phylogenetic trees and haplotype networks. Each color represents one clade, and hatched areas indicate overlap of two clades. Lists of samples analyzed in this study clustering with the each of the clades are provided using their unique codes (see Table S4 for accession numbers of the sequences).
